# Prevalence of chronic cough, its risk factors and population attributable risk in the Burden of Obstructive Lung Disease (BOLD) study: a multinational cross-sectional study

**DOI:** 10.1016/j.eclinm.2024.102423

**Published:** 2024-01-21

**Authors:** Hazim Abozid, Jaymini Patel, Peter Burney, Sylvia Hartl, Robab Breyer-Kohansal, Kevin Mortimer, Asaad A. Nafees, Mohammed Al Ghobain, Tobias Welte, Imed Harrabi, Meriam Denguezli, Li Cher Loh, Abdul Rashid, Thorarinn Gislason, Cristina Barbara, Joao Cardoso, Fatima Rodrigues, Terence Seemungal, Daniel Obaseki, Sanjay Juvekar, Stefanni Nonna Paraguas, Wan C. Tan, Frits M.E. Franssen, Filip Mejza, David Mannino, Christer Janson, Hamid Hacene Cherkaski, Mahesh Padukudru Anand, Hasan Hafizi, Sonia Buist, Parvaiz A. Koul, Asma El Sony, Marie-Kathrin Breyer, Otto C. Burghuber, Emiel F.M. Wouters, Andre F.S. Amaral, BOLD Collaborative Research Groupao, Hasan Hafizi, Anila Aliko, Donika Bardhi, Holta Tafa, Natasha Thanasi, Arian Mezini, Alma Teferici, Dafina Todri, Jolanda Nikolla, Rezarta Kazasi, Hamid Hacene Cherkaski, Amira Bengrait, Tabarek Haddad, Ibtissem Zgaoula, Maamar Ghit, Abdelhamid Roubhia, Soumaya Boudra, Feryal Atoui, Randa Yakoubi, Rachid Benali, Abdelghani Bencheikh, Nadia Ait-Khaled, Christine Jenkins, Guy Marks, Tessa Bird, Paola Espinel, Kate Hardaker, Brett Toelle, Michael Studnicka, Torkil Dawes, Bernd Lamprecht, Lea Schirhofer, Akramul Islam, Syed Masud Ahmed, Shayla Islam, Qazi Shafayetul Islam, Mesbah-Ul-Haque, Tridib Roy Chowdhury, Sukantha Kumar Chatterjee, Dulal Mia, Shyamal Chandra Das, Mizanur Rahman, Nazrul Islam, Shahaz Uddin, Nurul Islam, Luiza Khatun, Monira Parvin, Abdul Awal Khan, Maidul Islam, Herve Lawin, Arsene Kpangon, Karl Kpossou, Gildas Agodokpessi, Paul Ayelo, Benjamin Fayomi, Bertrand Mbatchou, Atongno Humphrey Ashu, Wan C. Tan, Wen Wang, NanShan Zhong, Shengming Liu, Jiachun Lu, Pixin Ran, Dali Wang, Jin-ping Zheng, Yumin Zhou, Rain Jogi, Hendrik Laja, Katrin Ulst, Vappu Zobel, Toomas-Julius Lill, Ayola Akim Adegnika, Tobias Welte, Isabelle Bodemann, Henning Geldmacher, Alexandra SchwedaLinow, Thorarinn Gislason, Bryndis Benedikdtsdottir, Kristin Jorundsdottir, Lovisa Gudmundsdottir, Sigrun Gudmundsdottir, Gunnar Gudmundsson, Mahesh Rao, Parvaiz A. Koul, Sajjad Malik, Nissar A. Hakim, Umar Hafiz Khan, Rohini Chowgule, Vasant Shetye, Jonelle Raphael, Rosel Almeda, Mahesh Tawde, Rafiq Tadvi, Sunil Katkar, Milind Kadam, Rupesh Dhanawade, Umesh Ghurup, Sanjay Juvekar, Siddhi Hirve, Somnath Sambhudas, Bharat Chaidhary, Meera Tambe, Savita Pingale, Arati Umap, Archana Umap, Nitin Shelar, Sampada Devchakke, Sharda Chaudhary, Suvarna Bondre, Savita Walke, Ashleshsa Gawhane, Anil Sapkal, Rupali Argade, Vijay Gaikwad, Sundeep Salvi, Bill Brashier, Jyoti Londhe, Sapna Madas, Althea Aquart-Stewart, Akosua Francia Aikman, Talant M. Sooronbaev, Bermet M. Estebesova, Meerim Akmatalieva, Saadat Usenbaeva, Jypara Kydyrova, Eliza Bostonova, Ulan Sheraliev, Nuridin Marajapov, Nurgul Toktogulova, Berik Emilov, Toktogul Azilova, Gulnara Beishekeeva, Nasyikat Dononbaeva, Aijamal Tabyshova, Kevin Mortimer, Wezzie Nyapigoti, Ernest Mwangoka, Mayamiko Kambwili, Martha Chipeta, Gloria Banda, Suzgo Mkandawire, Justice Banda, Li-Cher Loh, Abdul Rashid, Siti Sholehah, Mohamed C. Benjelloun, Chakib Nejjari, Mohamed Elbiaze, Karima El Rhazi, E.F.M. Wouters, G.J. Wesseling, Daniel Obaseki, Gregory Erhabor, Olayemi Awopeju, Olufemi Adewole, Amund Gulsvik, Tina Endresen, Lene Svendsen, Asaad A. Nafees, Muhammad Irfan, Zafar Fatmi, Aysha Zahidie, Natasha Shaukat, Meesha Iqbal, Luisito F. Idolor, Teresita S. de Guia, Norberto A. Francisco, Camilo C. Roa, Fernando G. Ayuyao, Cecil Z. Tady, Daniel T. Tan, Sylvia Banal-Yang, Vincent M. Balanag, Maria Teresita N. Reyes, Renato B. Dantes, Renato B. Dantes, Lourdes Amarillo, Lakan U. Berratio, Lenora C. Fernandez, Norberto A. Francisco, Gerard S. Garcia, Teresita S. de Guia, Luisito F. Idolor, Sullian S. Naval, Thessa Reyes, Camilo C. Roa, Flordeliza Sanchez, Leander P. Simpao, Ewa Nizankowska-Mogilnicka, Jakub Frey, Rafal Harat, Filip Mejza, Pawel Nastalek, Andrzej Pajak, Wojciech Skucha, Andrzej Szczeklik, Magda Twardowska, Cristina Barbara, Fatima Rodrigues, Herminia Dias, Joao Cardoso, João Almeida, Maria Joao Matos, Paula Simão, Moutinho Santos, Reis Ferreira, M. Al Ghobain, H. Alorainy, E. El-Hamad, M. Al Hajjaj, A. Hashi, R. Dela, R. Fanuncio, E. Doloriel, I. Marciano, L. Safia, Eric Bateman, Anamika Jithoo, Desiree Adams, Edward Barnes, Jasper Freeman, Anton Hayes, Sipho Hlengwa, Christine Johannisen, Mariana Koopman, Innocentia Louw, Ina Ludick, Alta Olckers, Johanna Ryck, Janita Storbeck, Kirthi Gunasekera, Rajitha Wickremasinghe, Asma Elsony, Hana A. Elsadig, Nada Bakery Osman, Bandar Salah Noory, Monjda Awad Mohamed, Hasab Alrasoul Akasha Ahmed Osman, Namarig Moham ed Elhassan, Abdel Mu’is El Zain, Marwa Mohamed Mohamaden, Suhaiba Khalifa, Mahmoud Elhadi, Mohand Hassan, Dalia Abdelmonam, Christer Janson, Inga Sif Olafsdottir, Katarina Nisser, Ulrike SpetzNystrom, Gunilla Hagg, GunMarie Lund, Terence Seemungal, Fallon Lutchmansingh, Liane Conyette, Imed Harrabi, Myriam Denguezli, Zouhair Tabka, Hager Daldoul, Zaki Boukheroufa, Firas Chouikha, Wahbi Belhaj Khalifa, Ali Kocabas, Attila Hancioglu, Ismail Hanta, Sedat Kuleci, Ahmet Sinan Turkyilmaz, Sema Umut, Turgay Unalan, Peter G.J. Burney, Anamika Jithoo, Louisa Gnatiuc, Hadia Azar, Jaymini Patel, Caron Amor, James Potts, Michael Tumilty, Fiona McLean, Risha Dudhaiya, A. Sonia Buist, Mary Ann McBurnie, William M. Vollmer, Suzanne Gillespie, Sean Sullivan, Todd A. Lee, Kevin B. Weiss, Robert L. Jensen, Robert Crapo, Paul Enright, David M. Mannino, John Cain, Rebecca Copeland, Dana Hazen, Jennifer Methvin

**Affiliations:** a Department of Respiratory and Pulmonary Diseases, Clinic Penzing, Vienna Healthcare Group, Vienna, Austria; b Ludwig Boltzmann Institute for Lung Health, Vienna, Austria; c National Heart and Lung Institute, Imperial College London, London, UK; d Sigmund Freud University, Faculty for Medicine, Vienna, Austria; e Department of Respiratory and Pulmonary Diseases, Clinic Hietzing, Vienna Healthcare Group, Vienna, Austria; f University of Cambridge, Cambridge, UK; g Liverpool University Hospitals NHS Foundation Trust, Liverpool, UK; h Department of Community Health Sciences, Aga Khan University, Karachi, Pakistan; i King Saud bin Abdulaziz University for Health Sciences, Riyadh, Saudi Arabia; j King Abdullah International Medical Research Centre, Riyadh, Saudi Arabia; k Department of Respiratory Medicine/Infectious Disease, Member of the German Centre for Lung Research, Hannover School of Medicine, Germany; l Ibn El Jazzar Faculty of Medicine of Sousse, University of Sousse, Sousse, Tunisia; m Department of Pneumology, Faculty of Medicine Annaba, University Badji Mokhtar of Annaba, Annaba, Algeria; n Royal College of Surgeons in Ireland and University College Dublin Malaysia Campus, Penang, Malaysia; o Faculty of Medicine, University of Iceland, Reykjavík, Iceland; p Department of Sleep, Landspitali - The National University Hospital of Iceland, Reykjavik, Iceland; q Instituto de Saúde Ambiental, Faculdade de Medicina, Universidade de Lisboa, Lisbon, Portugal; r Serviço de Pneumologia, Centro Hospitalar Universitário Lisboa Norte, Lisbon, Portugal; s Pulmonology Department, Centro Hospitalar Universitário de Lisboa Central, Lisboa, Portugal; t NOVA Medical School, Nova University Lisbon, Lisboa, Portugal; u Institute of Environmental Health, Associate Laboratory TERRA, Lisbon Medical School, Lisbon University, Lisbon, Portugal; v Faculty of Medical Sciences, University of West Indies, St Augustine, Trinidad and Tobago; w Department of Medicine, Obafemi Awolowo University, Ile-Ife, Nigeria; x Faculty of Medicine, University of British Columbia, Vancouver, Canada; y Vadu Rural Health Program, KEM Hospital Research Centre, Pune, India; z Philippine College of Chest Physicians, Manila, Philippines; aa University of British Columbia Centre for Heart Lung Innovation, St Paul's Hospital, Vancouver, BC, Canada; ab Maastricht University Medical Centre, Maastricht, the Netherlands; ac Centre for Evidence Based Medicine, 2nd Department of Internal Medicine, Jagiellonian University Medical College, Kraków, Poland; ad University of Kentucky, Lexington, KY, USA; ae COPD Foundation, Miami, FL, USA; af Department of Medical Sciences, Respiratory, Allergy and Sleep Research, Uppsala University, Uppsala, Sweden; ag Department of Pneumology, Faculty of Medicine Annaba, University Badji Mokhtar of Annaba, Annaba, Algeria; ah Department of Respiratory Medicine, JSS Medical College, JSSAHER, Mysuru, India; ai Faculty of Medicine, Tirana University Hospital “Shefqet Ndroqi”, Tirana, Albania; aj Oregon Health & Science University, Portland, USA; ak Department of Pulmonary Medicine, Sheri Kashmir Institute of Medical Sciences, Srinagar, India; al Epi-Lab, Khartoum, Sudan; am NIHR Imperial Biomedical Research Centre, London, UK

**Keywords:** Chronic cough, Epidemiology, Global health, Excess risk

## Abstract

**Background:**

Chronic cough is a common respiratory symptom with an impact on daily activities and quality of life. Global prevalence data are scarce and derive mainly from European and Asian countries and studies with outcomes other than chronic cough. In this study, we aimed to estimate the prevalence of chronic cough across a large number of study sites as well as to identify its main risk factors using a standardised protocol and definition.

**Methods:**

We analysed cross-sectional data from 33,983 adults (≥40 years), recruited between Jan 2, 2003 and Dec 26, 2016, in 41 sites (34 countries) from the Burden of Obstructive Lung Disease (BOLD) study. We estimated the prevalence of chronic cough for each site accounting for sampling design. To identify risk factors, we conducted multivariable logistic regression analysis within each site and then pooled estimates using random-effects meta-analysis. We also calculated the population attributable risk (PAR) associated with each of the identifed risk factors.

**Findings:**

The prevalence of chronic cough varied from 3% in India (rural Pune) to 24% in the United States of America (Lexington,KY). Chronic cough was more common among females, both current and passive smokers, those working in a dusty job, those with a history of tuberculosis, those who were obese, those with a low level of education and those with hypertension or airflow limitation. The most influential risk factors were current smoking and working in a dusty job.

**Interpretation:**

Our findings suggested that the prevalence of chronic cough varies widely across sites in different world regions. Cigarette smoking and exposure to dust in the workplace are its major risk factors.

**Funding:**

10.13039/100010269Wellcome Trust.

**Research in context**

**Evidence before this study**

We searched PubMed, from database inception to 30 July 2023, using terms such as “chronic cough,” “epidemiology,” “prevalence,” “risk factor,” “adult,” and “general population”. Prevalence estimates for chronic cough ranged from 2% to 18%. Smoking, asthma, obesity, chronic obstructive pulmonary disease (COPD), upper airway cough syndrome (UACS), gastro-oesophageal reflux disease (GORD), and low education level are some of the factors that have most commonly been associated with chronic cough. A systematic assessment of chronic cough and its risk factors across several world regions is lacking.

**Added value of this study**

This study provides population-based estimates of prevalence of chronic cough for 41 sites across several world regions using the same protocol and definition. It also provides a list of risk factors for chronic cough and the proportion of prevalence explained by each risk factor in each study site. Current smoking and working in a dusty job are the most influential risk factors for chronic cough worldwide. Besides a better understanding of the geographic variation in chronic cough prevalence and of the characteristics of people with chronic cough, a common finding across study sites and regions was that chronic cough may persist for several years.

**Implications of all the available evidence**

The available evidence should be used to better support public health strategies targeting chronic cough and attenuate its burden on global health.

## Introduction

Chronic cough (CC) is one of the most common reasons why people seek medical attention.^1^ Regardless of the underlying cause, CC has a significant impact on daily activities^2^ and is linked to poorer health status in general populations.^3^^,^^4^ It has been associated with psychosocial conditions, urinary incontinence, and depression,^5^^,^^6^ as well as higher healthcare use and cost.^7^ Yet, little is known about its true prevalence in various regions of the world.

A systematic review published in 2015 reported a wide range of prevalence estimates for CC across world regions, varying from 2.3% in Africa to 18.1% in Oceania.^8^ However, less than a third of the studies were conducted in Africa and Asia, studies were not primarily designed to assess CC, and the definition was not the same across the studies, which poses challenges in comparing data across different studies. Even in more recent studies and within the same country the definition of CC varies.^4^^,^^9^

Several factors have been associated with CC, but with some exceptions the list of its established risk factors is limited. Smoking, chronic obstructive pulmonary disease (COPD), asthma, upper airway cough syndrome (UACS), and gastro-oesophageal reflux disease (GORD) are suggested as the most common causes of CC.^10^, ^11^, ^12^, ^13^ Recent reviews emphasise the importance of conducting extensive epidemiological studies that can identify the prevalence and relevant risk factors in general populations,^11^ and address the need to utilise a standardised definition of CC in representative populations from different countries.^14^ In this context, our study is well-suited and effectively fulfills this crucial need. Using a standardised protocol, we aimed to provide estimates of prevalence of CC for several sites across the world. It was also our aim to identify the most important risk factors for CC.

## Methods

### Study design

A detailed description of the BOLD cohort has been published elsewhere.^15^ In brief, non-institutionalised adults (≥40 years old) were identified and recruited from the general population in 41 sites with more than 150,000 inhabitants. In each site, the aim was to recruit a minimum of 600 participants, with equal number of males and females. Sampling strategies varied across sites, with some using cluster sampling and others using either simple random sampling or stratified random sampling. For each site and participant, weights were derived to account for sampling design and to preserve representativeness of prevalence estimates.

The 41 study sites were located across 34 countries and several world regions: 11 in Europe (Tirana in Albania, Salzburg in Austria, London in England, Tartu in Estonia, Hannover in Germany, Reykjavik in Iceland, Maastricht in the Netherlands, Bergen in Norway, Krakow in Poland, Lisbon in Portugal, and Uppsala in Sweden); 14 in Asia (Guangzhou in China, Kashmir, Mumbai, Mysore and Pune in India, Chui and Naryn in Kyrgyztan, Penang in Malaysia, Karachi in Pakistan, Manila and Nampicuan-Talugtug in the Philippines, Riyadh in Saudi Arabia, Sri Lanka, and Adana in Turkey); 11 in Africa (Annaba in Algeria, Seme-Kpodji in Benin, Limbe in Cameroon, Blantyre and Chikwawa in Malawi, Fes in Marocco, Ife in Nigeria, Uitsig-Ravensmead in South Africa, Gezeira and Khartoum in Sudan, and Sousse in Tunisia); 2 in North America (Vancouver in Canada, and Lexington, KY, in the United States of America); 2 in the Caribbean (Jamaica, and Trinidad and Tobago); and 1 in Australia (Sydney). Fourteen sites were in high-income countries, while 27 were in low- or middle-income countries.

Information on respiratory symptoms, health status, and exposure to potential risk factors was collected by trained fieldworkers, who administered standardised questionnaires translated into the local language. While prevalence data on CC is presented for 33,983 individuals who completed the core questionnaire and had data on CC, risk factor analyses are based on data from 28,639 participants who additionally provided acceptable quality post-bronchodilator spirometry. Recruitment occurred between Jan 2, 2003 and Dec 12, 2016.

### Ethics

All sites received approval from their local ethics committee, and participants provided informed consent. The study was conducted as per good clinical practice (GCP) as well as local ethics regulations.

### Definition of chronic cough

CC was defined as a cough, without having a cold, on most days for at least three months each year. It was assessed using two questions: “Do you usually cough when you don't have a cold?” and “Do you cough on most days for as much as three months each year?”, and participants who affirmatively responsed to both were classified as resporting CC.

### Potential risk factors

Based on prior knowledge, we considered several potential risk factors for CC. These included age (in years), sex (males, females), and smoking status.^11^ The main question for smoking was “Have you ever smoked cigarettes? (‘Yes‘ means more than 20 packs of cigarettes in a lifetime or more than 1 cigarette each day for a year)”. Participants who responded with ‘No’ were classified as never smokers. In case of a ‘Yes’ response, a subsequent question asked at what age the participant had stopped smoking, if applicable. Participants who provided a numerical response were classified as former smokers, while those who did not provide an answer were categorised as current smokers. We also considered passive smoking (‘yes’ to the question whether anyone (other than the participant) had smoked a cigarette, pipe, or cigar in the participant's home during the past 2 weeks), body mass index (BMI; underweight: <18.5 kg m^−2^, normal weight: 18.5–24.9 kg m^−2^, overweight: 25.0–29.9 kg m^−2^, obese: ≥30.0 kg m^−2^), years worked in a dusty job (‘yes’ to “have you ever worked for a year or more in a dusty job?” and answer to “for how many years have you worked in a dusty job?”), education (based on ‘‘How many years of schooling have you completed?”), history of tuberculosis (‘yes’ to “has a doctor or health care provider ever told you that you had tuberculosis?”), and hypertension (‘yes’ to “has a doctor or health care provider ever told you that you had hypertension?”). We did not include the use of solid fuels as a factor in our analyses due to previous findings of the BOLD study, which showed no association with CC.^16^ Chronic airflow obstruction (CAO) was assessed using spirometry (EasyOneTM, ndd Medizintechnik AG, Zurich, Switzerland) and defined as post-bronchodilator forced expiratory volume in 1 s (FEV1) to forced vital capacity (FVC) less than the lower limit of normal (LLN) for age and sex, based on equations of the National Health and Nutrition Examination Survey (NHANES III).^17^

### Statistical analysis

We used multivariable logistic regression to identify factors associated with CC. Our regression model included all potential risk factors listed above (i.e., age, sex, smoking status, passive smoking, BMI, years worked in a dusty job, education, history of tuberculosis, hypertension, and chronic airflow obstruction). We estimated the adjusted odds ratio for each factor within each site, and then pooled site-specific estimates using random effects meta-analysis.^18^ We used the I^2^ statistic to summarise heterogeneity across sites. Results were considered significant if the p-value was <0.05. Prevalence estimates and regression analysis were corrected for sampling weights. For each of the identified risk factors, we estimated the population attributable risk (PAR), i.e., the excess prevalence of CC that can be attributed to the risk factor.^19^ Analyses were conducted using Stata v.16 (Stata Corp., College Station, TX, USA), and a user-written program to call OpenBUGS into Stata.^20^

### Role of the funding source

The funders of the study did not contribute to the study design, data collection, data analysis or writing of the manuscript. All authors had full access to the data and accept responsibility for the decision to submit for publication.

## Results

### Population characteristics

The mean age of participants was 55 years, with slightly more females (53.3%) than males, and the mean BMI was 26.5 kg m^−2^. About two thirds were never smokers, 18.9% were current smokers, and passive smoking was reported by 19.2%. About one third of the participants worked in a dusty job. The mean duration of working in a dusty job was 5.6 years. The mean duration of schooling was 9 years. History of tuberculosis was reported by 2.3% and hypertension by 25.3% (Table 1). For more details on the distribution of these characteristics please see Supplementary Table S1.

**Table 1:** Characteristics and comparison of the BOLD participants with and without chronic cough.

	With chronic cough (n = 3444)	Without chronic cough (n = 30,539)	Total (n = 33,983)
Age (years), mean (SD)	57 (13)	56 (11)	55 (11)
Sex, n (%)			
Females	1873 (54.4%)	16,255 (53.2%)	18,128 (53.3%)
Males	1571 (45.6%)	14,284 (46.8%)	15,855 (46.7%)
BMI (kg.m^−2^), mean (SD)	27.2 (6.2)	26.42 (5.6)	26.5 (5.7)
Smoking status, n (%)			
Current smokers	1068 (31.0%)	5353 (17.5%)	6421 (18.9%)
Ex-smokers	705 (20.5%)	5772 (18.9%)	6477 (19.1%)
Never smokers	1671 (48.5%)	19,414 (66.6%)	21,085 (62.0%)
Passive smoking, n (%)	885 (25.7%)	5595 (18.3%)	6480 (19.2%)
Ever worked in a dusty job, n (%)	1497 (43.5%)	9660 (31.6%)	11,157 (32.8%)
Duration of work in a dusty job (years), mean (SD)	8 (13)	5 (11)	6 (11)
Schooling (years), mean (SD)	8 (5)	9 (5)	9 (5)
History of tuberculosis, n (%)	153 (4.4%)	624 (2.0%)	777 (2.3%)
Hypertension, n (%)	1178 (34.2%)	7429 (24.3%)	8607 (25.3%)
FEV1/FVC (%), mean (SD)	73.7 (12.0)	78.1 (8.2)	77.7 (8.7)

BMI: Body mass index. FEV1/FVC: Forced expiratory volume in 1 s to the forced vital capacity ratio. SD: Standard deviation.

Participants with CC were older, were more likely current smokers and had more frequent exposure to passive smoking and a dusty job. They also showed a higher BMI, a lower FEV1/FVC ratio, a lower education level (less schooling years) and a higher proportion of self-reported hypertension and tuberculosis history (Table 1).

### Prevalence of chronic cough

**Fig. 1:**
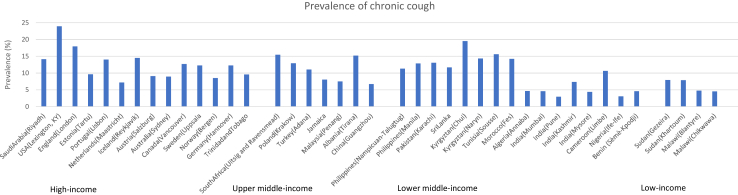
**Prevalence of chronic cough across 41 sites of the BOLD study, by gross national income**.

The pooled prevalence of CC in adults ≥ 40 years living in the study sites was 11.8%. However, variation across study sites was huge, ranging from 3% in India (rural Pune) to 24% in the United States of America (Lexington, KY). The lowest prevalence estimates were found in low- and middle-income countries (Fig. 1). The regions with the highest prevalence were North America (18.8%, 95% CI 16.5%–21%), Central Asia (18.4%, 95% CI 14.4%–22.5%), and Southern Africa (15.4%, 95% CI 12.6%–18.3%), whereas regions with the lowest prevalence were West Africa (3.6%, 95% CI 2.8%–4.3%), East Asia (6.7%, 95% CI 4.7%–8.7%), and East Africa (7.9%, 95% CI 6.5%–9.2%) (Table 2).

**Table 2:** Prevalence and characteristics of study participants with chronic cough across world regions, sorted by highest overall prevalence.

	North America	Central Asia	Southern Africa	Central & Eastern Europe	North Africa & Middle East	Western Europe	Central Africa	South East Asia	Australia	South Asia	Caribbean	East Africa	East Asia	West Africa
Chronic cough prevalence (%)	18.8 (16.5, 21)	18.4 (14.4, 22.5)	15.4 (12.6, 18.3)	15.1 (11.1, 19)	14.2 (11.5, 16.9)	12.6 (11.1, 14)	10.7 (7.3, 14)	9.9 (7.5, 12.3)	8.9 (6.6, 11.2)	8.9 (7, 10.9)	8.6 (7.3, 9.8)	7.9 (6.5, 9.2)	6.7 (4.7, 8.7)	3.6 (2.8, 4.3)
Participants with chronic cough (n)	247	314	135	275	488	696	45	393	52	304	194	176	40	85
Cough duration (%)														
<2 years	20.1 (14.8, 25.4)	20.8 (10.3, 31.3)	37.8 (28.8, 46.8)	19.1 (8.2, 29.9)	27.3 (15.4, 39.1)	21.2 (15.5, 26.8)	60.7 (43.7, 77.8)	54.8 (47.4, 62.3)	17.5 (6.6, 28.5)	28.5 (20.5, 36.6)	46 (36.9, 55.1)	20.1 (12.9, 27.4)	22.5 (8.8, 36.2)	46.9 (34.8, 58.9)
2–5 years	30.6 (24, 37.2)	31.8 (23.8, 39.7)	25.3 (17.1, 33.5)	50 (37.3, 62.8)	27.2 (20.1, 34.3)	19.2 (12.2, 26.3)	11.3 (1.6, 21)	24.7 (16.9, 32.4)	27.7 (15.1, 40.3)	31.8 (19.9, 43.6)	20.4 (11.2, 29.6)	33 (21.4, 44.5)	29.6 (15, 44.2)	15.5 (8.3, 22.6)
>5 years	49.3 (42.4, 56.2)	47.4 (42.4, 52.5)	36.9 (27.9, 46)	30.9 (21.8, 39.9)	45.5 (30.1, 60.9)	59.6 (52.1, 67.1)	28 (11.9, 44)	20.5 (14.5, 26.5)	54.8 (40.6, 69)	39.7 (29.9, 49.5)	33.6 (25.2, 42)	46.9 (37.3, 56.5)	48 (31.9, 64)	37.7 (25.8, 49.6)
Female (%)	52.3 (49.5, 55.2)	54.3 (48.1, 60.6)	56.1 (53.1, 59)	50.9 (48.2, 53.7)	50.6 (45.7, 55.5)	54.2 (48.2, 60.3)	40.6 (35, 46.2)	51.6 (48.7, 54.5)	52.9 (52.4, 53.5)	42.7 (40.8, 44.6)	51 (48.7, 53.3)	47 (42.2, 51.7)	49.4 (49.4, 49.4)	49.3 (46.4, 52.1)
Age group (%)														
40–49 years	34.2 (31.5, 37)	39.6 (36.2, 43)	43.7 (39.7, 47.7)	35.6 (32.7, 38.6)	44.4 (41, 47.8)	28.4 (24.3, 32.4)	50.9 (45.2, 56.6)	42.8 (39.3, 46.3)	31.8 (27.9, 35.7)	44.9 (41.8, 48.1)	36.3 (34.3, 38.4)	44.5 (41.6, 47.3)	44.9 (40.9, 48.8)	47.1 (44, 50.2)
50–59 years	28.3 (25.9, 30.6)	32.6 (29.1, 36.2)	29.5 (26, 33)	28.6 (24.8, 32.4)	26.9 (24.9, 29)	27.1 (25.4, 28.9)	27.8 (22.8, 32.7)	29.4 (26.5, 32.3)	25.7 (22.2, 29.2)	29.3 (26.5, 32)	29.1 (26.9, 31.3)	26.3 (24.1, 28.5)	25.1 (21.6, 28.6)	27 (24.7, 29.3)
60–69 years	17.8 (15.8, 19.8)	14.1 (10.1, 18)	17.8 (14.9, 20.7)	19.2 (16.5, 21.8)	16 (13.1, 18.9)	23.2 (20.9, 25.4)	17.7 (13.6, 21.7)	17.3 (15.6, 19)	18 (15.1, 20.9)	16.5 (14.4, 18.6)	18, 4 (16.7, 20.1)	15, 9 (14.1, 17.7)	19 (15.9, 22.2)	15 (13.4, 16.7)
70+ years	19.7 (17.1, 22.3)	13.7 (8.3, 19)	9 (6.8, 11.1)	16.6 (14.7, 18.4)	12.6 (11, 14.3)	21.3 (17.9, 24.8)	3.7 (1.8, 5.5)	10.5 (8, 12.9)	24.6 (20.9, 28.2)	9.3 (6.5, 12)	16.2 (14.7, 17.6)	13.3 (11, 15.7)	11 (8.5, 13.5)	10.9 (9, 12.8)
Education (%)														
None/primary school	2.9 (1.9, 3.9)	6 (2.9, 9)	44.7 (40.7, 48.6)	19.9 (15.3, 24.5)	69 (64.7, 73.3)	23.2 (20.5, 25.8)	57.1 (51.5, 62.8)	25.1 (22.2, 27.9)	3.6 (2, 5.1)	42.9 (39.3, 46.6)	30.7 (27.6, 33.8)	60.7 (54.6, 66.8)	28.9 (25.3, 32.5)	53.5 (50, 57)
High school	37.8 (35, 40.5)	50 (44.4, 55.6)	48.1 (44.3, 52)	45.1 (38.1, 52.2)	23.1 (19.1, 27.1)	47.2 (45.1, 49.3)	32 (26.7, 37.4)	62 (59.5, 64.5)	35.1 (31.2, 39)	36.7 (33.6, 39.7)	54.2 (51.3, 57.2)	28.5 (24.1, 32.8)	59.3 (55.4, 63.2)	31.1 (28.2, 34)
College/University	59.4 (56.6, 62.1)	44 (38.9, 49.2)	7.2 (5.2, 9.2)	35 (30.5, 39.4)	7.9 (3.7, 12)	29.6 (26.4, 32.8)	10.8 (7.4, 14.3)	12.9 (11.1, 14.7)	61.4 (57.4, 65.4)	20.4 (17.6, 23.2)	15.1 (12.8, 17.4)	10.9 (8.2, 13.6)	11.8 (9.2, 14.4)	15.5 (12.6, 18.3)
Smoking status (%)														
Never smokers	39.2 (36.4, 42)	63 (56.6, 69.4)	30.5 (27, 33.9)	63 (58.2, 67.7)	71.7 (67.4, 76)	48.6 (45.9, 51.3)	78.6 (73.9, 83.3)	62 (58.4, 65.5)	47.4 (43.3, 51.4)	81.2 (79, 83.4)	63.4 (60.4, 66.4)	76.6 (73.1, 80)	56.6 (53.9, 59.3)	89.6 (87.6, 91.5)
Current smokers	21.7 (19.3, 24.1)	26.7 (20.4, 33)	47.8 (44.1, 51.6)	22.3 (18.8, 25.8)	10.7 (7.7, 13.7)	20.4 (17.5, 23.2)	6.8 (4, 9.6)	26.5 (23.5, 29.5)	14.9 (12, 17.9)	11.8 (9.8, 13.7)	18.1 (15.6, 20.7)	8.8 (7.1, 10.5)	29.7 (26.6, 32.8)	3.4 (2.1, 4.7)
Ex-smokers	39.1 (36.3, 41.9)	10.3 (8.8, 11.8)	21.7 (18.7, 24.7)	14.8 (9.8, 19.7)	17.6 (15.1, 20)	31 (27.8, 34.3)	14.6 (10.5, 18.7)	11.5 (9.9, 13.1)	37.7 (33.8, 41.6)	7.1 (5.7, 8.4)	18.4 (16.5, 20.3)	14.6 (11.7, 17.5)	13.7 (11.1, 16.3)	7 (5.6, 8.5)
Passive smoking (%)	18.7 (16.5, 21)	7.2 (4.1, 10.4)	50 (45.7, 54.2)	36.7 (32.7, 40.7)	10.8 (8.9, 12.7)	18.8 (14.8, 22.8)	3.6 (1.3, 6)	35.4 (32.8, 38)	11 (8.5, 13.6)	7.1 (5.5, 8.6)	17.6 (15.6, 19.7)	9.1 (6.2, 12)	23 (19.7, 26.4)	1.2 (0.6, 1.7)
Body mass index (%)														
Underweight (<18.5 kg/m2)	0.3 (0, 0.6)	1.2 (0.6, 1.8)	7.1 (5.2, 9)	0.1 (0, 0.3)	1.8 (0.9, 2.6)	0.3 (0.1, 0.5)	1.1 (−0.1, 2.3)	4.7 (3.2, 6.2)	0.3 (−0.1, 0.8)	6.7 (5.2, 8.2)	3.5 (2.6, 4.5)	4.3 (2.9, 5.8)	4.7 (3, 6.4)	4 (2.9, 5.1)
Normal (18.5–24.9 kg/m2)	29.1 (26.7, 31.5)	35.5 (32.9, 38.1)	33.2 (29.7, 36.6)	28.6 (25.9, 31.3)	29.8 (23.8, 35.8)	30.9 (28.5, 33.2)	43.2 (37.6, 48.8)	45.7 (43, 48.4)	30.6 (26.7, 34.6)	50.6 (47.3, 54)	35.6 (33, 38.2)	40.8 (37.8, 43.9)	69 (65.3, 72.7)	49.7 (46.7, 52.7)
Overweight (25–29.9 kg/m2)	35.6 (32.8, 38.4)	32.9 (28.6, 37.2)	27 (23.8, 30.2)	40.4 (38.2, 42.7)	36.6 (33.3, 40)	44.2 (42.3, 46.1)	32.1 (26.8, 37.4)	35.5 (32.8, 38.2)	38.8 (34.7, 42.9)	28.9 (26, 31.8)	31.1 (29, 33.3)	31.8 (29, 34.7)	22.9 (19.5, 26.3)	28 (25.5, 30.4)
Obese (≥30 kg/m2)	35 (32.3, 37.7)	30.4 (25, 35.9)	32.8 (29.5, 36.1)	30.8 (28.2, 33.4)	31.8 (26.6, 36.9)	24.6 (22.1, 27)	23.6 (18.6, 28.6)	14.1 (11.7, 16.5)	30.2 (26.4, 34.1)	13.8 (11.8, 15.8)	29.7 (27.5, 31.9)	23 (20.8, 25.2)	3.4 (2, 4.9)	18.3 (16.3, 20.4)
Ever worked in a dusty job (%)	42.2 (39.4, 45)	27.8 (18.7, 36.9)	49 (45.5, 52.6)	62.5 (55.8, 69.2)	40.5 (37.1, 43.8)	39.6 (34, 45.3)	61.2 (55.6, 66.8)	42 (38.8, 45.3)	31.9 (28.2, 35.6)	19.9 (17.1, 22.6)	50.5 (48.1, 52.9)	28 (25.4, 30.7)	36.2 (32.3, 40.1)	38.2 (35.5, 40.8)
Chronic airflow obstruction (%)	14 (11.9, 16)	11.5 (9, 13.9)	19.6 (16.6, 22.6)	8.8 (6.1, 11.6)	8.2 (6.5, 9.8)	10 (8.3, 11.7)	4.3 (1.7, 6.9)	6.2 (4.5, 7.9)	11.1 (8.4, 13.8)	8.4 (6.4, 10.3)	7.7 (5.8, 9.5)	8.5 (6.5, 10.5)	7.9 (5.4, 10.3)	7.1 (5.5, 8.8)
History of tuberculosis (%)	2.4 (1.6, 3.3)	1 (0.1, 1.9)	15.2 (12.6, 17.8)	0.8 (0.2, 1.4)	1.4 (0.2, 2.5)	3.7 (2.8, 4.6)	0.9 (−0.2, 2.1)	4.6 (2.9, 6.3)	0.6 (0, 1.2)	0.7 (0.3, 1.1)	0.3 (0, 0.7)	0.6 (0.2, 1)	3.2 (1.8, 4.6)	0.5 (0.1, 0.8)
Hypertension (%)	36.4 (33.7, 39.2)	27.9 (23.8, 32)	37.4 (34, 40.9)	24 (22.2, 25.8)	29.1 (25, 33.3)	32.8 (30.7, 35)	9.8 (6, 13.5)	24.5 (21.7, 27.3)	32.7 (28.8, 36.5)	20.6 (18.3, 22.9)	31.7 (29.6, 33.8)	17 (13.8, 20.2)	16.7 (13.8, 19.7)	10.7 (8.2, 13.3)

Estimates were obtained through meta-analysis. 95% confidence intervals are given in brackets.

Sites included in each world region: ***North America***–Canada (Vancouver), USA (Lexington, KY); ***Central Asia***–Kyrgyztan (Chui), Kyrgyztan (Naryn); ***Southern Africa***–South Africa (Uitsig and Ravensmead); ***Central and Eastern Europe***–Albania (Tirana), Estonia (Tartu), Poland (Krakow); ***North Africa & Middle East***–Algeria (Annaba), Morocco (Fes), Saudi Arabia(Riyadh), Tunisia (Sousse), Turkey (Adana); ***Western Europe***–Austria (Salzburg), England (London), Germany (Hannover), Iceland (Reykjavik), Netherlands (Maastricht), Norway (Bergen), Portugal (Lisbon), Sweden (Uppsala); ***Central Africa***–Cameroon (Limbe); ***South East Asia***–Malaysia (Penang), Philippines (Manila), Philippines (Nampicuan-Talugtug), Sri Lanka; ***Australia***–Australia (Sydney); ***South Asia***–India (Mumbai), India (Mysore), India (Pune), India (Kashmir), Pakistan (Karachi); ***Caribbean***–Jamaica, Trinidad and Tobago; Africa East–China (Guangzhou); ***East Asia***–Malawi (Blantyre), Malawi (Chikwawa), Sudan (Gezeira), Sudan (Khartoum); ***West Africa***–Benin (Sèmè-Kpodji), Nigeria (Ife-Ife).

From all the participants with CC, slightly more than a quarter (27.4%) reported having CC for less than two years, 29% for two to five years, and 43.6% for over five years. The region with the highest proportion of participants experiencing cough for over five years, in relation to all participants reporting CC, was Western Europe (59.6%, 95% CI 52.1%–67.1%). Amongst study sites, Bergen in Norway had the highest proportion (70.7%).

The region with the highest proportion of females experiencing CC was Southern Africa (56.1%, 95% CI 53.1%–59%), whereas the lowest proportion was found in Central Africa (40.6%, 9%%CI 35%–46.2%).

Regions with a higher proportion of younger participants with CC were primarily located in Africa and Asia, where most participants with CC were under 49 years old. The highest proportion of participants with CC aged 70 years or over were found in regions with mainly high-income study sites, i.e., Australia (24.6%), Western Europe (21.3%, 95% CI 17.9%–24.8%) and North America (19.7%, 95% CI 17.1%–22.3%).

The proportion of participants with lower level of education (none or primary school) amongst those with CC was highest in North Africa/Middle East (69%, 95% CI 64.7%–73.3%), East Africa (60.7%, 95% CI 54.6%–66.8%), and Central Africa (57.1%, 95% CI 51.5%–62.8%). The proportion of participants with a college or university degree amongst those with CC was highest in Australia (61.4%), North America (59.4%, 95% CI 56.6%–62.1%), and Central Asia (44%, 95% CI 38.9%–49.2%).

The proportion of current smokers amongst participants with CC were highest in Southern Africa (47.8%, 95% CI 44.1%–51.6%), East Asia (29.7%, 95% CI 26.6%–32.8%), and Central Asia (26.7%, 95% CI 20.4%–33%). Passive smoking amongst participants with CC was highest in Southern Africa (50%, 95% CI 45.7%–54.2%).

The proportion of obesity amongst participants with CC was highest in North America (35%, 95% CI 32.3%–37.7%), Southern Africa (32.8%, 95% CI 29.5%–36.1%) and North Africa/Middle East (31.8%, 95% CI 26.6%–36.9%).

Central and Eastern Europe had the highest proportion of dusty job exposure (62.5%, 95% CI 55.8%–69.2%) amongst participants with CC.

Regarding self-reported doctor-diagnoses amongst participants with CC, the prevalence of hypertension was highest in Southern Africa (37.4%, 95% CI 34%–40.9%), North America (36.4%, 95% CI 33.7%–39.2%), and Western Europe (32.8%, 95% CI 30.7%–35%), whereas a history of tuberculosis was most prevalent in Southern Africa (15.2%, 95% CI 12.6%–17.8%) followed by South East Asia (4.6%, 95% CI 2.9%–6.3%) and Western Europe (3.7%, 95% CI 2.8%–4.6%).

### Factors associated with chronic cough

CC was associated with being a female, current smoking, passive smoking, working in a dusty job, obesity, lower education, tuberculosis as well as hypertension and airflow limitation (Table 3).

**Table 3:** Adjusted estimates for the association of chronic cough with several risk factors.

	OR (95% CI)	p value	I^2^ (χ2 heterogeneity p value)
Age/per 10 years	0.99 (0.92–1.08)	0.89	58.7 (<0.001)
Female sex	1.50 (1.29–1.75)	<0.001	42 (<0.001)
Current smoker	2.07 (1.73–2.48)	<0.001	44.2 (<0.001)
Ex-smoker	1.15 (0.95–1.39)	0.16	41.7 (<0.001)
Passive smoking	1.29 (1.13–1.47)	<0.001	20.1 (0.15)
Working in a dusty job	1.64 (1.47–1.84)	<0.001	23.3 (0.1)
Underweight	1.02 (0.80–1.30)	0.88	0 (0.73)
Overweight	1.06 (0.95–1.18)	0.28	0 (0.84)
Obesity	1.20 (1.07–1.35)	<0.001	34.1 (0.02)
Schooling (per year)	0.97 (0.96–0.99)	0.01	50.4 (<0.001)
History of tuberculosis	1.77 (1.30–2.40)	<0.001	42.6 (0.01)
Hypertension	1.25 (1.14–1.38)	<0.001	58.9 (<0.001)
Post-BD FEV1/FVC	0.96 (0.95–0.96)	<0.001	58.7 (<0.001)

Underweight: <18.5 kg m^−2^, overweight: 25.0–29.9 kg m^−2^, obese: ≥30.0 kg m^−2^. Post-BD FEV1/FVC: Post bronchodilator Forced expiratory volume in 1 s as a ratio of the forced vital capacity.

**Fig. 2:**
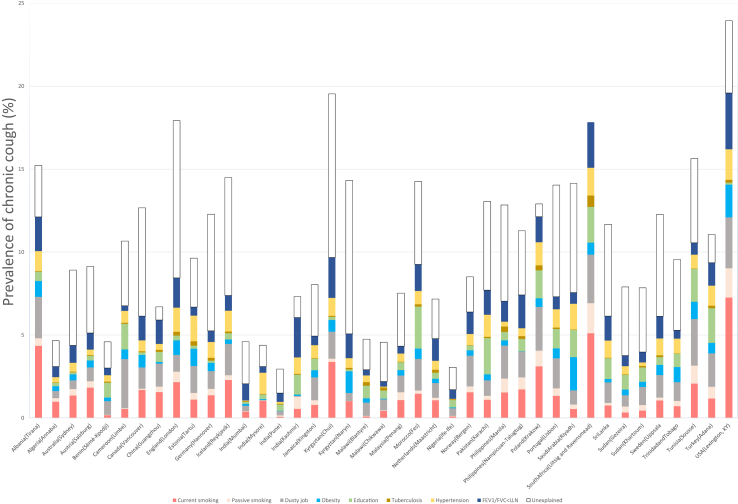
**Population Attributable Risk for chronic cough (i.e., prevalence of chronic cough attributable to different risk factors) by BOLD study site.** Each risk factor is represented by a different colour: red, current smoking; pink, passive smoking; grey, dusty job; light blue, obesity; green, education; brown, tuberculosis; yellow, hypertension; dark blue, FEV1/FVC < LLN; and white, unexplained.

Site specific PAR estimates for each risk factor associated with CC are shown in Fig. 2. Globally, current smoking was the most important risk factor (PAR 1.47%) followed by working in a dusty job (PAR 1.32%) (Table 4). Current smoking was the most important factor in South Africa (Uitsig and Ravensmead), where 33.1% of the CC prevalence can be explained by current smoking (current smoking PAR 5.1%; CC prevalence 15.4%). Working in a dusty job was most influent in Cameron (Limbe) with 27.9% (PAR 2.97%; prevalence 10.7%), followed by China (Guangzhou) and Norway (Bergen) with 21.7% of the prevalence of CC explained by this factor (PAR 1.85%; prevalence 8.51%). Kyrgyzstan (Naryn) had the highest proportion of unexplained prevalence of CC, where almost two-thirds (65.6%) of reported prevalence could not be attributed to a specific risk factor. The next highest proportions of unexplained prevalence were reported in Germany (Hannover) at 57.2% and India (Mumbai) at 55.1%.

**Table 4:** Risk factors for chronic cough ranked by population attributable risk, expressed as percent of total population aged ≥ 40 years, with 95% credible intervals.

	PAR	Lcrl	Ucrl
Current smoking	1.47	0.07	7.26
Dusty job	1.32	0.01	1.81
CAO	1.15	0.04	3.08
Hypertension	0.8	0.12	2.52
Education	0.78	0.02	2.01
Obesity	0.53	0.02	0.69
Passive smoking	0.4	0.02	1.85
Tuberculosis	0.11	−0.3	3.39

PAR: population attributable risk, Lcrl: Lower credible interval, Ucrl: Upper credible interval, CAO: chronic airflow obstruction.

## Discussion

Using real-world data, we report a pooled prevalence of 11.8% for CC, with significant variation across world regions and study sites. CC was associated with being a female as well as current smoking, passive smoking, working in a dusty job, a history of tuberculosis, obesity, lower education level, hypertension and lower FEV1/FVC. The most influential of these factors were current smoking and working in a dusty job.

Reported prevalence in general populations can be up to 18%^2^^,^^10^^,^^12^^,^^13^^,^^21^, ^22^, ^23^, ^24^ and is lower in Asia, with representative data reported from China and Korea.^3^^,^^4^^,^^9^^,^^13^ Of note, potential causes for variation in prevalence estimates are due to the use of different study designs, sample populations, definitions and ethnicities.^8^ In our study, using one definition for all study sites, the pooled prevalence of CC was 11.8%, with wide variation ranging from 3% in India (Pune) to 24% in the United States of America (Lexington, KY). Overall, we found that low- and middle-income sites had lower prevalence of CC, and that site specific findings about CC can not easily be extrapolated to country or region level. However, one common feature among individuals with CC is the experience of coughing for several years which has been reported previously in numerous studies.^2^^,^^21^^,^^25^, ^26^, ^27^, ^28^, ^29^ Our study underlines this burden by showing that more than 70% of all participants have experienced CC for more than two years, with almost half (44.7%) of them having experienced CC for more than 5 years.

Most data about risk factors in general populations derive from Europe or Asia.^11^^,^^14^ In Europe, the Rotterdam study has identified current smoking, GORD, asthma, and chronic obstructive pulmonary disease (COPD) as independent risk factors for CC in the general population.^10^ In another population study from Copenhagen, the analysis of risk factors was stratified by smoking status and showed that female sex, asthma, and GORD were associated with CC in never smokers, abdominal obesity, low income, and asthma were associated in ex-smokers, and airflow limitation in current smokers.^12^ In Asia, current smoking, older age, and UACS were identified as risk factors by a recent cross-sectional general population study of the Korean National Health and Nutrition Examination Survey.^3^^,^^4^ Although several conditions are often suggested in the clinical literature as potential risk factors, some of them may only have limited evidence in general populations.^11^ We report a strong association of current smoking with CC, and identified it as the most influential risk factor for CC worldwide. Current smoking has been frequently linked to CC^4^^,^^10^^,^^13^^,^^23^, ^24^, ^25^ but to our knowledge, population attributable risk analysis in CC has been only applied in one study, which also found that smoking is the most important risk factor.^12^ Our study has also identified working in a dusty job as another important risk factor contributing to CC worldwide. Data about dust as a risk factor of CC are scarce. In a Danish study, exposure to dust was ranked as only the seventh most influential factor.^12^ Other associations had been described earlier in Poland^30^ and in Singapore.^31^ Another study from Norway reported that men with high exposure to dust had a higher incidence of CC.^32^ Our findings emphasise the importance of recognizing distinct risk factors in different regions to effectively address the needs of each population and further develop specific prevention and management strategies.

Our data indicate a slightly higher female prevalence in CC compared to the non-CC group (54.4% vs 53.2%), but we were unable to confirm a significant difference. Regarding regional patterns, we found higher proportions of CC among males in almost all Asian regions, as well as in some African regions. Several European population based studies conducted in the UK,^33^ Germany,^23^ Austria,^25^ and Denmark^12^ reported more women but did not state a significant sex- or gender-specific difference. The Rotterdam study reported a female predominance for the age under 70 years but no significant sex-specific differences in the total study population.^10^ One of the assumed explanations for the female predominance is a heightened cough reflex.^34^, ^35^, ^36^ In contrast to this, a systematic review reported higher male prevalence in CC.^37^ More recent studies from general populations in China^4^ and Korea^3^^,^^13^ as well as from Canada^24^ reported more CC in males.

While current smoking was the most important risk factor, we were unable to detect an association of former smoking with CC, which is consistent with findings from the Rotterdam study and results from a meta-analysis.^10^^,^^11^ However, studies conducted in Germany and Austria found that former smoking was a significant risk factor for CC.^23^^,^^25^ For smoking exposure in terms of passive smoking, we found a positive association with CC as previously described in other studies.^38^^,^^39^

Obesity is a significant risk factor in our study and literature suggests a positive association between CC and obesity.^10^^,^^22^^,^^40^^,^^41^ It has been reported that people with obesity may be at higher risk of developing CC as compared to people without obesity.^10^^,^^24^ From the Copenhagen General Population Study, an up to threefold higher risk in people with obesity has been reported.^42^ A partial explanation for this increased risk was attributed to GORD, which mediated up to one quarter of CC cases in obesity. This finding provides a potential explanation for the relationship between obesity and CC, where an increased BMI is linked to an elevated risk of GORD.^43^ Therefore, practical guidelines for managing CC due to GORD recommend dietary modifications to promote weight loss in people with obesity.^44^ However, it is important to note that a direct relationship between GORD and CC could not be analysed in this study.

In our study, age is not a significant global risk factor for CC which contrasts with literature stating that CC is age-related and typically found in middle-aged to elderly people.^3^^,^^4^^,^^6^^,^^10^^,^^24^^,^^45^ It is important to note, however, that our study considers data from regions beyond Europe and Asia, providing a more comprehensive view of the global landscape of CC.

Our study reveals a link between CC and level of education. In contrast to a German study that did not find any significant difference in education levels,^23^ a Norwegian community cohort study reported that people with a lower educational level had a higher risk of developing CC.^46^ Similar findings outside Europe were observed in China^4^ and Nigeria.^47^ Additionally, the findings of a recent systematic review are in line with our results, indicating that individuals with lower levels of education are at a higher risk of developing CC.^11^ In this study, a history of tuberculosis was identified as a significant risk factor. Tuberculosis is not generally considered one of the most common causes of CC, even in high prevalence countries.^48^ A Korean study found that history of tuberculosis was more prevalent among people with CC.^13^ However, contrary to our findings, tuberculosis was not identified as a significant risk factor in the Korean general population.^3^

We observed a significantly higher prevalence of hypertension among individuals with CC. Additionally, we found that the presence of hypertension was associated with CC. Studies from Asian populations, particularly from China and Korea, reported significantly higher proportions of hypertension amongst people with CC.^4^^,^^13^ Similar results were also reported in Austria, where the association between CC and hypertension was significant as well.^25^ Within the context of practical guidelines,^49^^,^^50^ various cardiac diseases including those involving pulmonary congestion, are identified as potential causes of CC. In addition to chronic left heart failure and the use of cardiac drugs (like ACE inhibitors, beta-blockers, and Amiodarone), arrhythmias have also been reported in contributing to CC.^51^, ^52^, ^53^ Given that our study did not assess or investigate these specific conditions, it is challenging to draw conclusions regarding the role of hypertension alone in CC so that further cardiovascular phenotyping is required.

Our study has several strengths. First, it is a large comprehensive study covering several sites across several world regions, making it representative of populations. Given that our study included only adults over the age of 40, it should be noted that the findings cannot be extrapolated across all age groups in the population. Data collection was conducted using a standardised protocol and one definition of CC for all study sites. Data collection was undertaken by trained interviewers in local language. The study also has limitations. The main one is its cross-sectional nature, which prevents us from inferring causal relationships between risk factors and CC. We acknowledge that the definition we used differs from the definition stated in the most recent guidelines, which specify a cough lasting for a minimum of 8 weeks. We adopted the 3-month cut-off as this duration has been used in the majority of epidemiological studies.^8^^,^^37^ The prevalence of CC may be affected by recall bias as it was self-reported. Since the presence of cough is a subjective state that was not quantitatively measured in this study but evaluated through questionnaires, the differences in prevalence may also indicate variations in the perception and interpretation of cough across different cultures and regions. Our study did not include information about antihypertensive drugs to evaluate the proportion of ACE-inhibitors as potential triggers of iatrogenic CC. Additionally, we were unable to provide proportions of reflux cough as we did not collect information on GORD which could lead to potential overestimation of the unexplained CC prevalence in PAR analysis.

In summary, CC is common in many parts of the world. However, its prevalence varies considerably across regions, with low- and middle-income countries showing the lowest estimates of CC prevalence. Besides current smoking, exposure to dust in the workplace was identified as an important risk factor for CC. Our study emphasises the need for a better understanding of CC and its risk factors to provide tailored relief strategies for this troublesome condition.

## Contributors

HA, EFMW, and AFSA conceived the study. AFSA and JP accessed and verified the data. JP performed data analysis. Under the supervision of EFMW and AFSA, HA prepared the initial draft. All authors provided critical revision of the manuscript, as well as read and approved the final manuscript. All authors had full access to the data and accept responsibility for the decision to submit for publication.

## Data sharing statement

De-identified participant data and questionnaires may be shared, after publication, on a collaborative basis upon reasonable request made to Dr Amaral (a.amaral@imperial.ac.uk). Requesting researchers will be required to submit an analysis plan.

## Declaration of interests

Fatima Rodrigues declares grants and personal fees from A. Menarini, Boehringer Ingelheim, Teva Pharma, Novartis, GlaxoSmithKline, AstraZeneca, VitalAire and Nippon Gases outside the submitted work. Wan C. Tan received grants from the Canadian Institute of Heath Research (CIHR/Rx&D Collaborative Research Program Operating Grants- 93,326) with industry partners Astra Zeneca Canada Ltd., Boehringer-Ingelheim Canada Ltd, GlaxoSmithKline Canada Ltd, Merck, Novartis Pharma Canada Inc., Nycomed Canada Inc., Pfizer Canada Ltd. for conducting the longitudinal population-based Canadian Cohort of Obstructive Lung Disease (CanCOLD) study on COPD. David Mannino is a consultant to GSK, AstraZeneca, Regeneron, Genentech, COPD Foundation, and expert witness on behalf of people suing Tobacco Industry (Schlesinger Law Firm). Sonia Buist is Chair of the Data Safety & Monitoring Board for the RELIANCE Clinical Trial. Frits Franssen declares personal fees from AstraZeneca, Chiesi, GlaxoSmithKline, MSD, Pieris, and Verona Pharma. Robab Breyer-Kohansal declares consulting fees from AstraZeneca, Boehringer Ingelheim, GlaxoSmithKline, Menarini, Novartis Pharma, and Sanofi, and participation on advisory boards for AstraZeneca, Menarini, and Sanofi. Thorarinn Gislason received a grant from the Icelandic Research Fund. Kevin Mortimer declares participation on advisory boards for AstraZeneca and GlaxoSmithKline. Sylvia Hartl declares grants from GSK, Chiesi Farma, Menarini Pharma, and AstraZeneca, and participation on advisory boards for Menarini Pharma and GSK. AFSA declares a grant from the COLT Foundation (CF/01/21).

## Appendix ASupplementary data

Supplementary Table S1 and BOLD Group members
