# Supplementary material for: Prevalence of chronic cough, its risk factors and population attributable risk in the Burden of Obstructive Lung Disease (BOLD) study: a multinational cross-sectional study
Source: eClinicalMedicine. 2024 Jan 21;68:102423. doi: 10.1016/j.eclinm.2024.102423 (PMC10807979; doi:10.1016/j.eclinm.2024.102423)
Supplement: Supplementary Table S1 and BOLD Group members [file mmc1.pdf]

Supplementary Table S1. Baseline characteristics of participants in the BOLD baseline study, per site.

			Age group				Highest level of education			Smoking status			BMI								
**Site**	**N**	**Female**	**40-49 yrs**	**50-59 yrs**	**60-69 yrs**	**70+ yrs**	**None / primary**	**High school**	**College / University**	**Never**	**Current**	**Ex**	**Normal**	**Underweight**	**Overweight**	**Obese**	**Passive smoking**	**Dusty job**	**CAO**	**TB**	**Hypertension**
Albania (Tirana)	997	50·7	35·9	28·7	19·1	16·3	20·3	45·1	34·5	63·2	22·4	14·4	28·6	0·1	40·5	30·7	37·3	63·3	8·9	0·6	23·6
Algeria (Annaba)	917	49·9	42·0	31·0	15·8	11·2	43·6	36·2	20·2	61·1	17·3	21·6	29·7	1·3	35·1	34·0	10·9	28·7	6·4	2·2	22·7
Australia (Sydney)	583	52·8	31·7	25·8	18·0	24·5	3·6	35·0	61·4	47·3	14·9	37·8	30·6	0·3	38·7	30·3	11·0	31·9	11·1	0·6	32·6
Austria (Salzburg)	1,349	54·1	27·1	24·9	23·8	24·2	13·4	63·3	23·3	48·1	19·9	32·0	39·6	0·7	41·7	17·9	21·8	26·0	17·4	3·1	32·0
Benin (Sèmè-Kpodji)	846	51·2	53·8	26·1	13·8	6·36	74·7	20·6	4·7	97·8	1·9	0·3	43·4	2·9	29·9	23·8	0·1	35·6	7·3	0·6	28·9
Cameroon (Limbe)	388	40·6	50·9	27·8	17·7	3·68	57·1	32·0	10·8	78·6	6·8	14·6	43·2	1·1	32·1	23·6	3·6	61·2	4·3	1·0	9·8
Canada (Vancouver)	855	51·8	35·9	27·5	15·7	20·8	2·6	21·9	75·6	42·0	15·2	42·9	42·0	0·4	35·8	21·8	5·9	30·6	13·4	3·1	21·0
China (Guangzhou)	602	49·4	44·9	25·1	19·0	11·0	28·9	59·3	11·8	56·6	29·7	13·7	69·0	4·7	22·9	3·4	23·0	36·2	7·9	3·2	16·8
England (London)	672	53·7	32·4	25·8	19·3	22·5	6·3	38·7	55·0	33·6	22·6	43·9	37·7	0·3	38·3	23·8	16·7	26·3	17·7	2·0	36·0

			Age group				Highest level of education			Smoking status			BMI								
**Site**	**N**	**Female**	**40-49 yrs**	**50-59 yrs**	**60-69 yrs**	**70+ yrs**	**None / primary**	**High school**	**College / University**	**Never**	**Current**	**Ex**	**Normal**	**Underweight**	**Overweight**	**Obese**	**Passive smoking**	**Dusty job**	**CAO**	**TB**	**Hypertension**
Estonia (Tartu)	647	60·5	28·3	23·7	23·2	24·9	2·7	44·7	52·7	55·0	17·8	27·1	28·3	0·4	37·6	33·7	15·6	34·4	6·1	7·3	40·0
Germany (Hannover)	713	54·1	28·3	26·5	28·9	16·3	1·8	65·2	33·0	38·0	23·8	38·1	32·4	1·0	45·4	21·1	18·8	23·2	8·2	3·7	38·0
Iceland (Reykjavik)	760	48·8	34·1	29·1	17·7	19·1	9·2	29·0	61·8	34·1	21·2	44·7	28·3	0·4	43·4	27·9	16·7	31·4	11·3	4·9	32·9
India (Mumbai)	515	40·9	41·1	30·2	18·0	10·7	25·4	47·3	27·3	91·5	5·5	2·9	61·6	5·9	25·3	7·2	1·2	6·0	6·6	0·6	10·7
India (Mysore)	724	56·8	75·1	15·5	7·8	1·6	21·5	45·7	32·9	89·1	10·2	0·7	54·0	3·3	33·4	9·2	0·0	2·4	8·0	0·0	19·6
India (Pune)	1,387	48·2	42·3	25·9	19·1	12·7	64·2	29·9	5·8	88·8	7·8	3·4	63·3	17·0	17·1	2·6	9·6	21·4	6·1	0·9	6·2
India (Kashmir)	952	49·4	54·3	22·4	15·2	8·0	82·2	15·1	2·7	48·4	44·6	7·1	70·1	8·3	16·6	5·0	64·4	0·4	16·1	0·5	26·0
Jamaica	795	51·3	37·0	28·1	17·6	17·3	24·6	63·4	12·1	60·6	18·6	20·8	39·0	4·4	28·6	28·0	16·4	56·4	8·4	0·5	33·1
Kyrgyztan (Chui)	1,031	54·9	39·2	33·0	14·4	13·4	4·8	54·1	41·0	60·8	28·9	10·3	34·6	1·1	32·4	31·9	8·2	33·7	12·5	1·1	31·0
Kyrgyztan (Naryn)	1,063	52·3	41·0	31·5	12·7	14·8	10·3	34·4	55·2	71·2	18·6	10·2	38·7	1·3	34·8	25·2	3·6	5·9	7·8	0·7	16·0

			Age group				Highest level of education			Smoking status			BMI								
**Site**	**N**	**Female**	**40-49 yrs**	**50-59 yrs**	**60-69 yrs**	**70+ yrs**	**None / primary**	**High school**	**College / University**	**Never**	**Current**	**Ex**	**Normal**	**Underweight**	**Overweight**	**Obese**	**Passive smoking**	**Dusty job**	**CAO**	**TB**	**Hypertension**
Malawi (Blantyre)	583	47·1	53·4	26·5	13·1	7·0	53·5	42·0	4·5	85·1	4·2	10·6	55·0	5·4	24·1	15·5	2·4	26·7	6·9	5·1	20·2
Malawi (Chikwawa)	795	51·2	42·8	25·1	19·0	13·1	91·4	8·1	0·4	68·9	19·8	11·3	73·3	12·7	10·0	4·0	3·5	24·0	12·9	4·8	3·3
Malaysia (Penang)	713	50·6	39·6	30·2	18·4	11·8	35·3	57·4	7·3	74·8	20·6	4·6	42·1	2·6	38·7	16·6	26·6	32·3	3·4	0·0	24·5
Morocco (Fes)	966	51·0	43·4	25·8	16·5	14·3	73·5	20·9	5·6	71·4	10·7	17·9	32·3	2·0	37·0	28·7	11·4	43·6	8·9	1·3	29·5
Netherlands (Maastricht)	630	52·5	29·1	27·5	20·3	23·1	13·5	32·4	54·1	35·2	24·8	40·0	29·1	0·1	46·6	24·2	17·9	23·6	18·8	1·3	30·5
Nigeria (Ife-Ife)	1,148	48·2	43·8	27·5	15·6	13·1	43·1	36·2	20·7	85·6	4·1	10·3	52·8	4·5	27·0	15·7	1·6	39·5	7·0	0·4	2·0
Norway (Bergen)	707	51·9	31·8	27·1	18·4	22·6	7·9	54·2	37·9	35·7	28·2	36·0	37·9	0·6	43·7	17·7	21·7	39·5	11·8	0·2	27·7
Pakistan (Karachi)	1,040	44·2	48·0	28·6	15·2	8·2	59·3	26·8	14·0	71·5	17·6	11·0	39·1	7·4	32·8	20·7	12·6	33·0	10·6	0·9	29·8
Philippines (Manila)	918	52·8	47·6	27·8	15·6	9·1	11·4	67·4	21·2	44·1	35·1	20·8	49·8	7·0	31·9	11·3	49·2	55·4	9·4	11·0	24·7
Philippines (Nampicuan-Talugtug)	984	53·9	38·0	29·3	19·0	13·8	22·8	64·0	13·2	46·3	35·5	18·2	68·0	16·0	14·5	1·5	48·4	35·8	15·0	5·1	24·5

			Age group				Highest level of education			Smoking status			BMI								
**Site**	**N**	**Female**	**40-49 yrs**	**50-59 yrs**	**60-69 yrs**	**70+ yrs**	**None / primary**	**High school**	**College / University**	**Never**	**Current**	**Ex**	**Normal**	**Underweight**	**Overweight**	**Obese**	**Passive smoking**	**Dusty job**	**CAO**	**TB**	**Hypertension**
Poland (Krakow)	603	51·0	35·2	25·0	21·1	18·6	38·7	47·5	13·9	39·9	27·9	32·2	30·1	0·7	40·6	28·6	37·8	49·4	13·4	3·4	43·4
Portugal (Lisbon)	744	55·0	28·2	27·3	22·3	22·2	37·1	39·7	23·2	56·3	18·6	25·1	27·4	0·0	44·4	28·2	19·1	51·0	8·3	4·5	32·2
Saudi Arabia (Riyadh)	784	48·0	51·4	35·2	12·4	1·0	36·9	38·8	24·3	74·7	10·0	15·3	12·4	0·4	33·8	53·5	5·5	17·1	3·2	1·8	27·2
South Africa (Uitsig and Ravensmead)	893	56·1	43·8	29·5	17·8	9·0	44·6	48·2	7·2	30·4	47·9	21·7	33·1	7·1	26·9	32·9	50·1	48·9	19·6	15·2	37·4
SriLanka	1,152	52·5	38·6	35·9	19·6	6·0	28·2	69·6	2·3	76·1	15·5	8·3	48·1	7·6	32·4	11·9	8·1	37·3	8·4	1·0	22·2
Sudan (Gezeira)	816	49·9	41·7	25·8	16·8	15·7	66·9	25·5	7·6	75·7	8·5	15·8	43·3	2·8	29·4	24·6	12·1	29·8	5·2	0·4	13·2
Sudan (Khartoum)	595	45·2	46·7	26·5	15·3	11·5	56·3	30·4	13·3	77·2	8·9	13·8	38·9	5·3	33·5	22·3	7·2	27·0	10·2	0·7	19·6
Sweden (Uppsala)	587	52·8	22·5	32·4	22·3	22·7	14·1	37·0	48·8	41·8	15·5	42·8	36·4	0·1	42·9	20·5	6·3	30·9	9·6	1·0	29·2
Trinidad and Tobago	1,381	50·7	35·1	30·9	19·9	14·2	41·7	37·7	20·6	68·6	17·3	14·2	29·7	2·0	35·6	32·7	19·9	39·9	6·3	0·1	29·2
Tunisia (Sousse)	717	49·4	48·3	28·8	16·3	6·5	51·5	38·0	10·5	55·5	31·0	13·5	23·1	1·5	37·5	37·9	34·6	49·2	5·3	0·0	17·9

Site	N	Female	Age group: 40-49 yrs	50-59 yrs	60-69 yrs	70+ yrs	Highest level of education: None / primary	High school	College / University	Smoking status: Never	Current	Ex	BMI: Normal	Underweight	Overweight	Obese	Passive smoking	Dusty job	CAO	TB	Hypertension
Turkey (Adana)	868	51·3	42·7	29·8	16·5	11·0	79·1	17·5	3·4	45·4	34·4	20·1	19·6	0·8	36·0	43·6	54·3	50·8	14·4	2·3	26·7
USA (Lexington, KY)	563	52·8	32·8	28·9	19·5	18·8	3·2	51·2	45·6	36·9	27·2	35·9	18·1	0·3	35·4	46·2	29·6	52·0	14·4	1·9	49·5

CAO: Chronic airflow obstruction; TB: History of tuberculosis

BOLD (Burden of Obstructive Lung Disease) Collaborative Research Group members

Albania: Hasan Hafizi (principal investigator [PI]), Anila Aliko, Donika Bardhi, Holta Tafa, Natasha Thanasi, Arian Mezini, Alma Teferici, Dafina Todri, Jolanda Nikolla, and Rezarta Kazasi (Tirana University Hospital Shefqet Ndroqi, Albania); Algeria: Hamid Hacene Cherkaski (PI), Amira Bengrait, Tabarek Haddad, Ibtissem Zgaoula, Maamar Ghit, Abdelhamid Roubhia, Soumaya Boudra, Feryal Atoui, Randa Yakoubi, Rachid Benali, Abdelghani Bencheikh, and Nadia Ait-Khaled (Faculte de M edecine Annaba, Service de Epidemiologie et M edecine Preventive, El Hadjar, Algeria); Australia: Christine Jenkins (PI), Guy Marks (PI), Tessa Bird, Paola Espinel, Kate Hardaker, and Brett Toelle (Woolcock Institute of Medical Research, Sydney, Australia); Austria: Michael Studnicka (PI), Torkil Dawes, Bernd Lamprecht, and Lea Schirhofer (Department of Pulmonary Medicine, Paracelsus Medical University, Salzburg, Austria); Bangladesh: Akramul Islam (PI), Syed Masud Ahmed (Co-PI), Shayla Islam, Qazi Shafayetul Islam, Mesbah-Ul-Haque, Tridib Roy Chowdhury, Sukantha Kumar Chatterjee, Dulal Mia, Shyamal Chandra Das, Mizanur Rahman, Nazrul Islam, Shahaz Uddin, Nurul Islam, Luiza Khatun, Monira Parvin, Abdul Awal Khan, and Maidul Islam (James P. Grant School of Public Health, BRAC [Building Resources Across Communities] University, Institute of Global Health, Dhaka, Bangladesh); Benin: Herve Lawin (PI), Arsene Kpangon, Karl Kpossou, Gildas Agodokpessi, Paul Ayelo, and Benjamin Fayomi (Unit of Teaching and Research in Occupational and Environmental Health, University of Abomey Calavi, Cotonou, Benin); Cameroon: Bertrand Mbatchou (PI) and Atongno Humphrey Ashu (Douala General Hospital, Douala, Cameroon); Canada: Wan C. Tan (PI) and Wen Wang (iCapture Center for Cardiovascular and Pulmonary Research, University of British Columbia, Vancouver, BC, Canada); China: NanShan Zhong (PI), Shengming Liu, Jiachun Lu, Pixin Ran, Dali Wang, Jin-ping Zheng, and Yumin Zhou (Guangzhou Institute of Respiratory Health, First Affiliated Hospital of Guangzhou Medical College, Guangzhou, China); Estonia: Rain Jogi (PI), Hendrik Laja, Katrin Ulst, Vappu ~ Zobel, and Toomas-Julius Lill (Lung Clinic, Tartu University Hospital, Tartu, Estonia); Gabon: Ayola Akim Adegnika (PI) (Centre de Recherches Medicale de Lambarene, Lambarene, Gabon); Germany: Tobias Welte (PI), Isabelle Bodemann, Henning Geldmacher, and Alexandra Schweda-Linow (Department of Pneumology, Hannover Medical School and German Center of Lung Research, Hannover, Germany); Iceland: Thorarinn Gislason (PI), Bryndis Benedikdtsdottir, Kristin Jorundsdottir, Lovisa € Gudmundsdottir, Sigrun Gudmundsdottir, and Gunnar Gudmundsson (Department of Allergy, Respiratory Medicine, and Sleep, Landspitali University Hospital, Reykjavik, Iceland); India: Mahesh Rao (PI) (JSS Medical College, Mysuru, India); Parvaiz A. Koul (PI), Sajjad Malik, Nissar A. Hakim, and Umar Hafiz Khan (Sher-i-Kashmir Institute of Medical Sciences, Srinagar, J&K, India); Rohini Chowgule (PI), Vasant Shetye, Jonelle Raphael, Rosel Almeda, Mahesh Tawde, Rafiq Tadvi, Sunil Katkar, Milind Kadam, Rupesh Dhanawade, and Umesh Ghurup (Indian Institute of Environmental Medicine, Mumbai, India); Sanjay Juvekar (PI), Siddhi Hirve, Somnath Sambhudas, Bharat Chaidhary, Meera Tambe, Savita Pingale, Arati Umap, Archana Umap, Nitin Shelar, Sampada Devchakke, Sharda Chaudhary, Suvarna Bondre, Savita Walke, Ashleshsa Gawhane, Anil Sapkal, Rupali Argade, and Vijay Gaikwad (Vadu Health and Demographic Surveillance System, King Edward Memorial Hospital Research Centre Pune, Pune India); Sundeep Salvi (PI), Bill Brashier, Jyoti Londhe, and Sapna Madas (Chest Research Foundation, Pune India); Jamaica: Althea Aquart-Stewart (PI) and Akosua Francia Aikman (University of the West Indies, Kingston, Jamaica); Kyrgyzstan: Talant M. Sooronbaev (PI), Bermet M. Estebesova, Meerim Akmatalieva, Saadat Usenbaeva, Jypara Kydyrova, Eliza Bostonova, Ulan Sheraliev, Nuridin Marajapov, Nurgul Toktogulova, Berik Emilov, Toktogul Azilova, Gulnara Beishekeeva, Nasyikat Dononbaeva, and AijamalTabyshova (Pulmonology and Allergology Department, National Centre of Cardiology and Internal Medicine, Bishkek, Kyrgyzstan); Malawi: Kevin Mortimer (PI), Wezzie Nyapigoti, Ernest

Mwangoka, Mayamiko Kambwili, Martha Chipeta, Gloria Banda, Suzgo Mkandawire, and Justice Banda (the Malawi Liverpool Wellcome Trust, Blantyre, Malawi); Malaysia: Li-Cher Loh (PI), Abdul Rashid, and Siti Sholehah (Royal College of Surgeons in Ireland and University College Dublin Malaysia Campus); Morocco: Mohamed C. Benjelloun (PI), Chakib Nejjari, Mohamed Elbiaze, and Karima El Rhazi (Laboratoire d'epid emiologie, Recherche Clinique et Sante Communautaire, F es, Morroco); Netherlands: E. F. M. Wouters and G. J. Wesseling (Maastricht University Medical Center, Maastricht, the Netherlands); Nigeria: Daniel Obaseki (PI), Gregory Erhabor, Olayemi Awopeju, and Olufemi Adewole (Obafemi Awolowo University, Ile-Ife, Nigeria); Norway: Amund Gulsvik (PI), Tina Endresen, and Lene Svendsen (Department of Thoracic Medicine, Institute of Medicine, University of Bergen, Bergen, Norway); Pakistan: Asaad A. Nafees (PI), Muhammad Irfan, Zafar Fatmi, Aysha Zahidie, Natasha Shaukat, and Meesha Iqbal (Aga Khan University, Karachi, Pakistan); Philippines: Luisito F. Idolor (PI), Teresita S. de Guia, Norberto A. Francisco, Camilo C. Roa, Fernando G. Ayuyao, Cecil Z. Tady, Daniel T. Tan, Sylvia Banal-Yang, Vincent M. Balanag, Jr., Maria Teresita N. Reyes, and Renato. B. Dantes (Lung Centre of the Philippines, Philippine General Hospital, Nampicuan and Talugtug, the Philippines); Renato B. Dantes (PI), Lourdes Amarillo, Lakan U. Berratio, Lenora C. Fernandez, Norberto A. Francisco, Gerard S. Garcia, Teresita S. de Guia, Luisito F. Idolor, Sullian S. Naval, Thessa Reyes, Camilo C. Roa, Jr., Ma. Flordeliza Sanchez, and Leander P. Simpao (Philippine College of Chest Physicians, Manila, the Philippines); Poland: Ewa Nizankowska-Mogilnicka (PI), Jakub Frey, Rafal Harat, Filip Mejza, Pawel Nastalek, Andrzej Pajak, Wojciech Skucha, Andrzej Szczeklik, and Magda Twardowska, (Division of Pulmonary Diseases, Department of Medicine, Jagiellonian University School of Medicine, Krakow, Poland); Portugal: Cristina Barbara (PI), Fatima Rodrigues, Herminia Dias, Joao Cardoso, João Almeida, Maria Joao Matos, Paula Simão, Moutinho Santos, and Reis Ferreira (the Portuguese Society of Pneumology, Lisbon, Portugal); Saudi Arabia: M. Al Ghobain (PI), H. Alorainy (PI), E. El-Hamad, M. Al Hajjaj, A. Hashi, R. Dela, R. Fanuncio, E. Doloriel, I. Marciano, and L. Safia (Saudi Thoracic Society, Riyadh, Saudi Arabia); South Africa: Eric Bateman (PI), Anamika Jithoo (PI), Desiree Adams, Edward Barnes, Jasper Freeman, Anton Hayes, Sipho Hlengwa, Christine Johannisen, Mariana Koopman, Innocentia Louw, Ina Ludick, Alta Olckers, Johanna Ryck, and Janita Storbeck, (University of Cape Town Lung Institute, Cape Town, South Africa); Sri Lanka: Kirthi Gunasekera (PI) and Rajitha Wickremasinghe (Medical Research Institute, Central Chest Clinic, Colombo, Sri Lanka); Sudan: Asma Elsony (PI), Hana A. Elsadig, Nada Bakery Osman, Bandar Salah Noory, Monjda Awad Mohamed, Hasab Alrasoul Akasha Ahmed Osman, Namarig Moham ed Elhassan, Abdel Mu'is El Zain, Marwa Mohamed Mohamaden, Suhaiba Khalifa, Mahmoud Elhadi, Mohand Hassan, and Dalia Abdelmonam (the Epidemiological Laboratory, Khartoum, Sudan); Sweden: Christer Janson (PI), Inga Sif Olafsdottir, Katarina Nisser, Ulrike SpetzNystrom, Gunilla Hagg, and Gun-Marie Lund (Department of Medical Sciences: Respiratory Medicine and Allergology, Uppsala University, Uppsala, Sweden); Trinidad and Tobago: Terence Seemungal (PI), Fallon Lutchmansingh, and Liane Conyette (University of the West Indies, St. Augustine, Trinidad and Tobago); Tunisia: Imed Harrabi (PI), Myriam Denguezli, Zouhair Tabka, Hager Daldoul, Zaki Boukheroufa, Firas Chouikha, and Wahbi Belhaj Khalifa (University Hospital Farhat Hached, Faculte de M edecine, Sousse, Tunisia); Turkey: Ali Kocabas¸ (PI), Attila Hancioglu, Ismail Hanta, Sedat Kuleci, Ahmet Sinan Turkyilmaz, Sema Umut, and Turgay Unalan (Department of Chest Diseases, Cukurova University School of Medicine, Adana, Turkey); UK: Peter G. J. Burney (PI), Anamika Jithoo, Louisa Gnatiuc, Hadia Azar, Jaymini Patel, Caron Amor, James Potts, Michael Tumilty, Fiona McLean, and Risha Dudhaiya (National Heart and Lung Institute, Imperial College London, London, UK); United States: A. Sonia Buist (PI) (Oregon Health & Science University, Portland, Oregon); Mary Ann McBurnie, William M. Vollmer, and Suzanne Gillespie (Kaiser Permanente

Center for Health Research, Portland, Oregon); Sean Sullivan (University of Washington, Seattle, Washington); Todd A. Lee and Kevin B. Weiss (Northwestern University, Chicago, Illinois); Robert L. Jensen and Robert Crapo (Latter Day Saints Hospital, Salt Lake City, Utah); Paul Enright (University of Arizona, Tucson, Arizona); David M. Mannino (PI), John Cain, Rebecca Copeland, Dana Hazen, and Jennifer Methvin (University of Kentucky, Lexington, Kentucky).
